# Mental fatigue in Olympic combat sports: the unseen ongoing battle

**DOI:** 10.3389/fspor.2025.1697860

**Published:** 2025-11-13

**Authors:** Chao Bian, Rafael Lima Kons, Kevin De Pauw, Marc Theeboom, Hebe Schaillée, Daniele Detanico, Bart Roelands

**Affiliations:** 1 Human Physiology and Sports Physiotherapy Research Group (MFYS), Vrije Universiteit Brussel, Brussels, Belgium; 2 Department of Physical Education, Faculty of Education, Federal University of Bahia, Salvador, Brazil; 3 Brussels Human Robotic Research Center (BruBotics), Vrije Universiteit Brussel, Brussels, Belgium; 4 Sport and Society Research Group (SASO), Vrije Universiteit Brussel, Brussels, Belgium; 5 Centre for Martial Arts and Science (CEMAS), Vrije Universiteit Brussel, Brussels, Belgium; 6 Biomechanics Laboratory, Federal University of Santa Catarina, Florianopolis, Brazil

**Keywords:** cognitive performance, cumulative fatigue, fencing, work-to-rest ratio, decision-making

## 1 Introduction

Combat sports, including judo, boxing, wrestling, taekwondo, and fencing, are popular worldwide and key to the upcoming 2028 Summer Olympics (1). Due to their growing relevance, research on these Olympic combat sports has expanded in recent years, focusing on physical (e.g., neuromuscular) and psychological (e.g., motivation) parameters (2–4). One-on-one combat typically involves high physical and cognitive demands (5). Generally, Olympic combat sports competitions consist of multiple matches held on the same day, often separated by short intervals/transitions (6). Meanwhile, the work-to-rest ratio (W/R) varies according to the specific group of the combats, reflecting differences in intensity patterns across striking, grappling, and weapon-based disciplines, from the perspective of performance and assessment (38). Table 1 presents the W/R for Olympic combat sports by discipline and group, ranging approximately from 1:9 to 9:1. Combined with the short rest time from official competitions [e.g., average 16.1 s between two pauses in the world championship Sabre bout (7)], these values emphasize the high intensity inherent in these sports with a very brief time window to recover. The highly intermittent, but prolonged competition format (i.e., congested matches in one day) suggests the buildup of fatigue in athletes, making it a key factor to be considered to actively manage on mat, ring, and piste.

**Table 1:** Work-to-rest ratio of Olympic combat sports.

Olympic combat sports	Groups	W/R	References
Taekwondo	Striking	1:3–1:4	Heller et al. (39)
Boxing		9:1	Davis et al. (33)
Judo	Grappling	2:1–3:1	Miarka et al. (34, 35)
Wrestling (Greco-Romano)		2.5:1	Nilsson et al. (36)
Wrestling (Freestyle)		2.4:1.	Sciranka et al. (37)
Fencing Épée	Weapon-based	1:0.9	Tarrago et al. (7)
Fencing Foil		1:2.6	
Fencing Sabre		1:9.2	

W/R, work-to-rest ratio.

Evidence has shown that the elevated intensity can result in significant physical fatigue accumulation throughout competition in different combat sports, as an example judo (3, 8, 9), taekwondo (10), and wrestling (11, 12). A systematic review conducted by Kons et al. (6) verified the effects of matches on physical fatigue in different combat sports and showed a decrease in physical performance in the upper and lower limbs, due to cumulative fatigue. However, these results were more evident in the grappling combat sport group, while striking modalities exhibited a different pattern, suggesting distinct behaviors between the two groups.

The physical aspect of combat sports has been extensively researched. However, despite recognition that fatigue is multidimensional (13) and important for athletic performance, the mental/cognitive counterpart (i.e., mental fatigue and cognitive performance) remains underexplored, especially given the different time structures across disciplines (Table 1). This is particularly relevant as athletes must make rapid decisions during attacks and counterattacks (14), anticipate opponents' actions within technical-tactical behaviors (15), and execute complex motor skills in unpredictable, high-pressure environments (4, 16). Olympic combat sports require strong perceptual-cognitive skills such as visual tracking (17, 18), selective attention (19), and inhibitory control (20), which are critical for maintaining strategic focus and effective decision-making under pressure (17). Moreover, athletes must manage high cognitive loads related to both environmental and situational demands, including adapting tactics, interpreting an opponent's actions, responding to coaches, ignoring distractions, and monitoring match status (e.g., score, time, and number of matches) (17). These challenges engage executive functions, which select, regulate, and coordinate behaviors in dynamic scenarios. Consequently, mental fatigue may arise from the sustained cognitive effort in combat (40). At the elite level, performance depends not only on physical capacity but also on efficient allocation of limited mental resources during critical moments (17, 21).

Although mental fatigue has been shown to impair physical, psychomotor, technical, and tactical performance in a variety of sports (40, 41), its actual presence and effects in Olympic combat sports have been largely overlooked. This is surprising given the high perceptual-cognitive demands these disciplines characterize (17) and their essential role in a competition success. In this context, the present opinion paper aims to explore the current state of research on mental fatigue in some Olympic combat sports (e.g., judo, taekwondo and fencing), highlighting existing evidence and proposing future directions for investigation. Specifically, it discusses how mental fatigue can impact both training and competitive settings in different Olympic combat sports. Furthermore, this paper contributes to the broader discourse beyond physical performance, emphasizing their potential in enhancing neurocognitive demands and mental effort imposed by matches and training. In this regard, this opinion paper highlights an underexplored perspective in combat sports research by drawing attention to the significant cognitive demands inherent in match situations and emphasizing the need for a deeper understanding of their implications on the performance of Olympic combat sport athletes.

## 2 Discussion

Few investigations explored the effects of mental fatigue on performance in Olympic combat sports. In general, studies used the computerized Stroop task, social media exposure, and specific demand for combat (e.g., quantify during official competition) to verify performance outcomes (21–27). Campos et al. (22) explored the effects of mental fatigue on judo athletes’ performance, aiming to verify whether cognitive exertion influenced physical, physiological, and perceptual responses during a specific judo test (*Special Judo Fitness Test*). Considering two experimental situations: one under controlled conditions and the other following the inducement of mental fatigue via a 30-minute Stroop task. The results showed increased perceptions of fatigue and effort, but no effect on judo-specific test performance or physiological responses, suggesting that mental fatigue did not significantly impact judo-specific test performance in judo athletes. However, it is important to emphasize that the study verified the effect of this situation on a specific judo test, which, despite reflecting some demands similar to judo (28, 29), does not reproduce its complexity (e.g., coaching instructions, noises, and monitoring match status) (17). Furthermore, the study's small and varied sample size may have impacted responses.

In taekwondo, Faro et al. (23) examined whether social media use induces mental fatigue comparable to a modified Stroop task, and whether either condition affects neuroelectric activity or sport-specific visuomotor performance in taekwondo athletes. A total of fifteen athletes were randomly allocated to two experimental conditions (social media use vs. Stroop task) and a control (watching a documentary). The modified Stroop task led to elevated mental fatigue on the visual analogue scale (VAS), slower reaction time, and reduced accuracy, along with changes in event-related potential components, indicating impaired cognitive processing. The social media use did not significantly affect fatigue or neuroelectric markers, and no difference was found for visual-motor performance related to the taekwondo-specific task in all conditions. Taking into account the specificity of taekwondo, this study brings a relevant point related to visual-motor performance, important for carrying out specific actions (e.g., kicks) (30, 42). However, it does not reproduce the real situations in which taekwondo athletes are subjected to in competition (10), and does not simulate the W/R of the matches.

In fencing, a higher number of studies have been carried out over the last few years. Varesco et al. (21) simulated a 5-bout competition in the laboratory with fourteen French national team fencers across three weapons. Repeated self-reports indicated that their gradual subjective manifestation of fatigue throughout the competition was linked to a significantly increased mental demand. The study advocated that the measured fatigue in fencing could have a strong cognitive component, traditionally referred to as “mental fatigue”. Located in real-world fencing, the survey of Bian et al. (24) directly targeted the mental fatigue concept among 92 Chinese national to world-level fencers. These elite fencers acknowledged the presence and broad impacts of mental fatigue in daily training and competition, with a highlight of the eliminatory stage in official competitions compared with the training sessions and preliminary stage matches. The contextual difference necessitated more convincing evidence from various actual fencing scenarios. Following up, Bian et al. (25) conducted a longitudinal tracking of mental fatigue in 39 Chinese national championship participants throughout 4 weeks, including preparation training, competition, recovery training, and regular training. The subjective (rating on VAS) and behavioral (performance in 3-minute psychomotor vigilance test) measurements aligned in competition days demonstrated the increased mental fatigue and its negative aftereffect on the fencer's reaction. The training structures, but not the training phase, showed an association with mental fatigue fluctuation. Further looking into the evolution of mental fatigue perceptions with consecutive match progression during one individual competition day, a clear accumulation trend has been captured (26). These studies suggest timely mental fatigue management and countermeasures in the early periods of the competitions and the tournament events, as well as in daily training by modulating cognitive demands for athletes’ mental fatigue adaptation and resilience (25). Relevantly, Varesco et al. (27) introduced 5 weeks of brain endurance training (BET) on top of the pre-season training schedule in French U17 and U20 national team. The BET intervention has been shown to benefit these youth elite fencers, as their steady cognitive performance following the Stroop task indicates strengthened adaptability in a mentally fatigued state.

## 3 Future perspectives

Currently, few studies have been performed towards the impact of mental fatigue in Olympic combat sports, except for fencing, where further research has been conducted. Further investigations should be conducted into other combat sports (e.g., wrestling, judo and boxing) and from a perspective that approximates an actual training/competition situation. In this aspect, future studies should verify the impacts of mental fatigue in Olympic combat sports using ecologically valid protocols that replicate the specific demands of competition (31), such as W/R, real-time decision-making under pressure, and cognitive/environmental demands during official matches. The on-field longitudinal tracking of mental fatigue with subjective and behavioral indicators across these combat sports, as well as the nighttime monitoring with other recovery indexes, should be conducted to develop better training schedules and long-term competition preparation. Additionally, aspects such as pre- and intra-competition activities, rapid weight loss strategies, inter-individual training/injury history, and the application of cognitive training (e.g., the volume and the timing of BET), which have demonstrated great implications in combat sports interventions (27, 32–37), should be considered to provide a more comprehensive understanding of Olympic combat sports performance under mental fatigue. Moreover, a key limitation in current research is the predominant reliance on the Stroop task and laboratory-based settings, which may reduce ecological validity and limit the applicability of findings to real-world competition scenarios. Finally, understanding sport-related aspects that most induce mental fatigue in individual sports, as combat modalities, may help athletes and coaches to adopt strategies to improve mental health and performance throughout the athlete's career.
